# Are anthropometric data a tool for determining the severity of OHSS? Yes, it could be!

**DOI:** 10.1186/s12905-022-01701-5

**Published:** 2022-05-10

**Authors:** Aleksei Petrovich Petrenko, Camil Castelo-Branco, Dimitry Vasilevich Marshalov, Alexander Valerievich Kuligin, Efim Munevich Shifman, Elena Sergeevna Nesnova, Batsunova Mariia Olegovna

**Affiliations:** 1 Clinic Institute of Gynecology, Obstetrics and Neonatology, Faculty of Medicine, University of Barcelona, Hospital Clinic-Institut d´Investigacions Biomèdiques August Pi i Sunyer (IDIBAPS), Villarroel 170, 08036 Barcelona, Spain; 2 Department of Emergency Anesthesiology-Resuscitation Care and Simulation Technologies in Medicine, Saratov State Medical University named after V. I. Razumovsky, Saratov, Russian Federation; 3 Department of Anesthesiology and Critical Care, State Budgetary Healthcare Institution of Moscow Region M.F. Vladimirsky Moscow’s Regional Research Clinical Institute, Moscow, Russian Federation; 4 City Clinical Hospital №1 named after Yu.Ya. Gordeev, Saratov, Russian Federation; 5 Department of Hospital Surgery, Saratov State Medical University named after V. I. Razumovsky, Saratov, Russian Federation

**Keywords:** Ovarian hyperstimulation syndrome, Anthropometic indicators, Intra-abdominal pressure, Intra-abdominal hypertension, Ascites index, Compliance of the abdominal wall

## Abstract

**Background:**

All management guidelines of ovarian hyperstimulation syndrome (OHSS) recommend daily monitoring of women's body weight, waist circumference and note that as indicators increase, the severity OHSS also increases. However, the dynamics of abdominal size and its relationship with markers of OHSS severity have not been highlighted. The purpose of this study is to assess the usefulness of various anthropometric indicators for determining the degree of OHSS severity as well as paracentesis indications.

**Methods:**

Observational study including 76 women complaining with OHSS. Clinical history, physical examination, laboratory tests, and ultrasound measurement of the ovarian volume (OV) and ascites index (AsI) were done in all cases. Intra-abdominal pressure (IAP) was assessed using an intravesical manometer. The anteroposterior diameter of the abdomen (APD) and transverse diameter of the abdomen (TS) were measured with a pelvimeter. The APD/TS ratio was calculated.

**Results:**

The APD/TS ratio increased progressively and tended to be the highest in the most symptomatic stage of OHSS (Kruskal–Wallis test, *p* < 0.001). The median APD/TS was significantly lower in patients with mild OHSS (0.55 [IQR, 0.44–0.64]) compared with severe OHSS (0.87 [IQR, 0.80–0.93]; *p* < 0.001) or critical OHSS (1.04 [IQR, 1.04–1.13]; *p* < 0.001). Similarly, the median APD/TS of the moderate OHSS group (0.65 [IQR, 0.61–0.70]) was significantly lower than that of the severe (*p* < 0.001) and critical OHSS group (*p* = 0.001). There was a strong positive correlation between APD/TS and IAP (Spearman’s *r* = 0.886, *p* < 0.01). The APD/TS ratio showed a significant positive correlation with AsI (Spearman’s *r* = 0.695, *p* < 0.01) and OV (Spearman’s *r* = 0.622, *p* < 0.01). No significant differences were observed in age, height, weight, body mass index, hip circumference or waist circumference between moderate, severe and critical OHSS groups.

**Conclusions:**

The APD/TS ratio is related to the severity of OHSS. Monitoring APD/TS dynamics could be a method of indirectly controlling intra-abdominal volume, compliance of the abdominal wall and IAP. In conjunction with clinical and laboratory data, APD/TS might be an indicator for paracentesis.

**Supplementary Information:**

The online version contains supplementary material available at 10.1186/s12905-022-01701-5.

## Background

Ovarian hyperstimulation syndrome (OHSS) is a largely iatrogenic condition, associated with significant morbidity and even mortality of healthy women undergoing fertility treatment [1, 2]. Generally, OHSS is triggered by human chorionic gonadotropin (hCG) and it's mainly due to excessive ovarian secretion of vascular endothelial growth factor and other angiogenic factors, increasing vascular permeability and causing fluid leakage into the third space [3, 4]. Thus, OHSS is characterized by enlarged ovaries with hypovolemia and haemoconcentration, in more severe cases including ascites, hypercoagulation, renal failure and even multiple organ failure in the critical ones [2]. The main principles in moderate and severe OHSS treatment are correction of hypovolemia, electrolyte imbalance, hypoalbuminemia and paracentesis, if necessary [5].

Ascites progression and ovarian enlargement with OHSS leads to an increase in intra-abdominal pressure (IAP), and in severe and critical formsto the abdominal compartment syndrome (ACS) and associated severe organ dysfunction, which is the main factor of poor outcome among women with this syndrome [6, 7]. Our previous study revealed OHSS as a classic model of intra-abdominal hypertension (IAH) syndrome, where IAP is an important diagnostic marker, allied with the OHSS severity [8]. It has been proposed, there was provided to use the IAH level and ascites index (AsI), for paracentesis's indications in combination with clinical and laboratory data. The IAP measuring through a Foley catheter by using a pressure transducer is the gold standard [9], but, unfortunately, it has not yet become widespread in gynecological and obstetric practice. Finding a simpler and more convenient method for indirect controlling intra-abdominal volume (IAV), abdominal wall compliance (Cab) and IAP without the use independently of complex and expensive techniques would be useful for OHSS management.

All OHSS management guidelines recommend daily monitoring of women's body weight, waist circumference (WC) and note that as indicators increase, the severity of OHSS also increases [5, 10–12]. However, according to the literature data, the dynamics of abdominal size and its relationship with markers of OHSS severity have not been highlighted.

The purpose of this study is to assess the usefulness of various anthropometric indicators in determining degree of OHSS severity as well as indications for paracentesis.

## Methods

### Sample

A total of 76 infertile women who were in an in vitro fertilization program and presented OHSS were included in this study. Sample size was established based on the fact that according to the Ministry of Health of the Saratov Region, during the period from 2015 to 2019, 4800 cycles of ART were performed in all medical institutions of the region. Complications presented by various forms of OHSS requiring outpatient monitoring and hospitalization, were recorded in 95 cases (1.9%). Thus, using the statistical software to calculate the sample size with a 5% maximum acceptable error, 95% confidence level, we obtained a sample size of 76 women with OHSS. All of them were admitted into the gynaecological department of the city clinical hospital No.1 named after Yu.Ya. Gordeev (Saratov, Russian Federation). Anthropometrical, laboratory and clinical data were recorded in all included subjects (Additional file 1: Table S1, Additional file 2: Table S2 and Additional file 3:  Table S3). The age range of the study participants was from 20 to 40 years old and the body mass index (BMI) was from 16.9 to 24.1 kg/m^2^.

OHSS was classified according to the Royal College of Obstetricians & Gynaecologists guidelines [5]. Therefore, patients were allocated into four groups depending on the severity of OHSS: mild OHSS (group I, n = 25), moderate OHSS (group II, n = 25), severe OHSS (group III, n = 21), and critical OHSS (group IV, n = 5). Early-onset OHSS was defined when the syndrome was initiated during the first 9 days after trigger administration of hCG, and late OHSS was defined when the syndrome was initiated from 10 days after. The current study included 19 (25%) women with early OHSS and 57 (75%) women with late OHSS. The IAP was measured 4 [IQR, 3–5] days after hCG administration in case of early OHSS and 17 [IQR, 13–19] days after hCG triggering in case of late OHSS. The average length of stay for subjects with early OHSS was 10 [IQR, 7–12] days; the average length of a hospital stay for women with late OHSS was 9 [IQR, 7–11] days. All women admitted with the diagnosis of OHSS were considered for inclusion in the study. Those who voluntarily refused to participate were excluded.

### Procedures

Anthropometrical and clinical data were recorded in all included subjects (Additional file 2: Table S2 and Additional file 3: Table S3). The anteroposterior diameter of the abdomen (APD) and transverse diameter of the abdomen (TS) were measured with a pelvimeter. The APD was defined as the distance between the spine at the L_3–4_ level and the abdomen apex, then the pelvimeter branches were rotated in the same plane, set along the midaxillary lines, and after that, TS measurement was made. The APD/TS ratio was calculated.

BMI was evaluated by the Quetelet’s equation, and in all cases blood and urine samples were obtained. Ovarian size and pelvic and abdominal free fluid were assessed by ultrasound (Accuvix XG [Samsung MEDISON Co. Ltd. Korea]) using 3.5 MHz sectoral sensors. The ovarian volume (OV) using the prolate ellipsoid formula [13] and the AsI [14] was measured as previously described [8]. Finally, the IAP was determined using a Foley catheter with a pressure transducer [9].

### Statistical analysis

The data were analysed using a personal computer-based software package (SPSS 26.0, SPSS Inc. Headquarters, 233 South Wacker Drive, 11th Floor, Chicago, IL 60606, USA). The Shapiro–Wilk test was used to determine the normal distribution of the sample. Data for non-normally distributed variables are given as the median [interquartile range]. Homogeneity of within-group variances was evaluated by Levene's test. The Kruskal–Wallis test was used to analyse differences between groups. Statistically significant results were followed by Mann–Whitney U-tests with Bonferroni adjustment to detect subgroup differences. Spearman’s correlation coefficients were used to check the association between continuous variables. All probability tests were two-sided and a *p*-value of < 0.05 was considered significant.

## Results

Anthropometrical data are given in Additional file 3: Table  S3. The age range of the study participants was from 20 to 40 years old and the BMI was from 16.9 to 24.1 kg/m^2^.

Significant differences between groups were observed regarding APD measurements (*p* < 0.001). The median APD of the mild OHSS group (16 [IQR, 15–19]) was found to be significantly lower than that of the severe (24 [IQR, 23–27], *p* < 0.001) and critical OHSS group (26 [IQR, 24–28], *p* = 0.001). Besides that, the median APD of the moderate OHSS group (19 [IQR, 17–24]) was significantly lower than that of the severe (*p* < 0.005) and critical OHSS group (*p* < 0.05). However, there was no significant difference in APD between mild and moderate or severe and critical OHSS groups (*p* > 0.05).

**Fig. 1:**
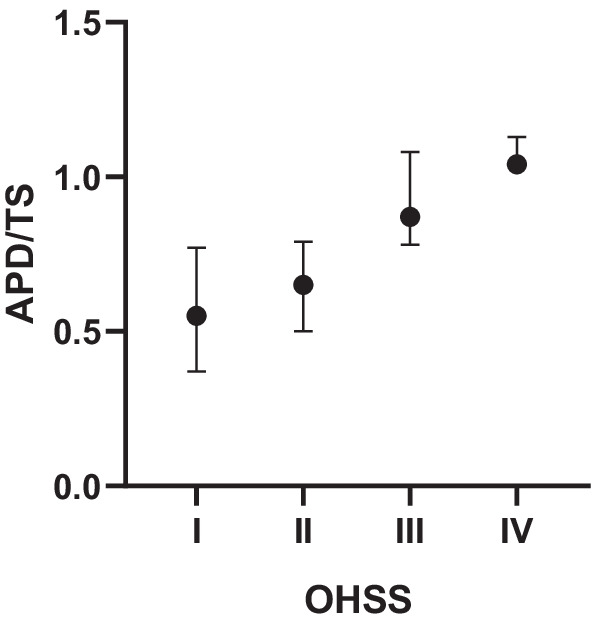
APD/TS according to severity of ovarian hyperstimulation syndrome. Data are plotted as median with range

As expected, APD/TS increased progressively and tended to be the highest in the most symptomatic stage of OHSS (*p* < 0.001). Figure 1 represents the intergroup comparison of APD/TS. The median APD/TS was significantly lower in patients with mild OHSS (0.55 [IQR, 0.44–0.64]) compared with severe OHSS (0.87 [IQR, 0.80–0.93]; *p* < 0.001) or critical OHSS (1.04 [IQR, 1.04–1.13]; *p* < 0.001). Similarly, the median APD/TS of the moderate OHSS group (0.65 [IQR, 0.61–0.70]) was significantly lower than that of the severe (*p* < 0.001) and critical OHSS group (*p* = 0.001). There was no significant difference in APD/TS between mild and moderate or severe and critical OHSS groups (*p* > 0.05).

No significant differences were observed in age, height, weight, body mass index, hip circumference or waist circumference between moderate, severe and critical OHSS groups (Additional file 2: Table S2). There was also no significant difference between the early and late OHSS groups (p > 0.05).

**Fig. 2:**
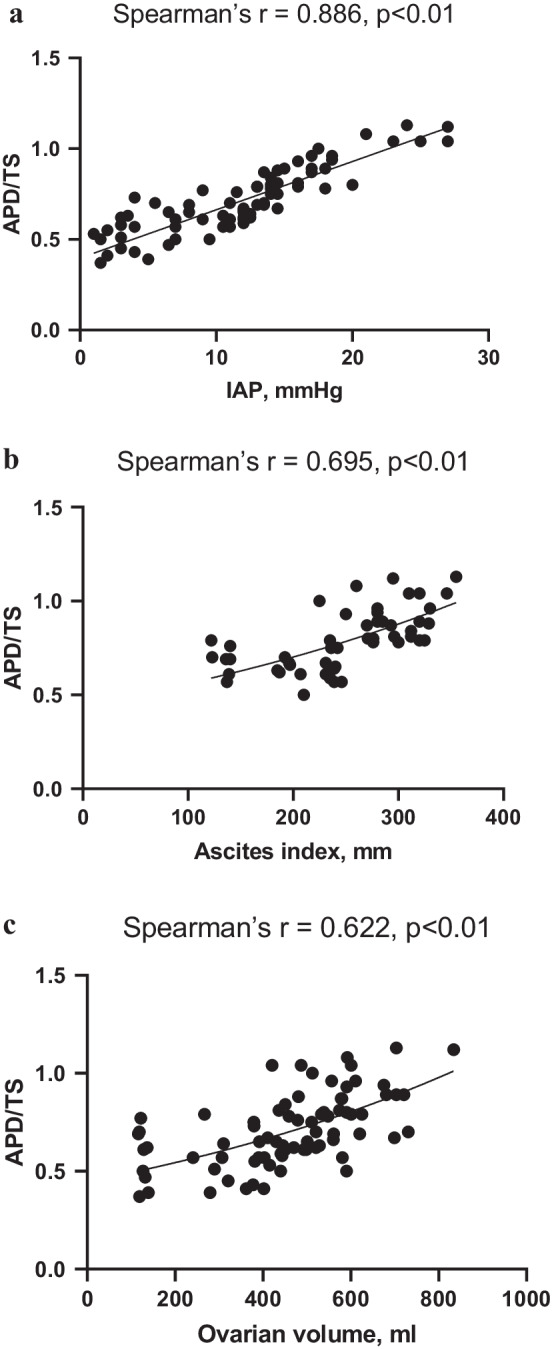
Scatter plots of APD/TS related to intra-abdominal pressure (**a**), ascites index (**b**) and ovarian volumes (**c**)

Correlation analysis was used to identify whether the APD/TS was independently associated with other anthropometric indicators and IAP, AsI or OV. As anticipated, there was a strong positive correlation between APD/TS and IAP (Spearman’s *r* = 0.886, *p* < 0.01; Fig. 2a). Besides that, APD/TS showed a significant positive correlation with AsI (Spearman’s *r* = 0.695, *p* < 0.01; Fig. 2b) and OV (Spearman’s *r* = 0.622, *p* < 0.01; Fig. 2c). No significant correlation was present between APD/TS and any of the other anthropometric indicators, except for a weak inverse correlation with WC (Spearman’s *r* = −0.24, *p* < 0.05). A significant but weak inverse correlation was observed between APD/TS and the age (Spearman’s *r* = −0.285, *p* < 0.05).

## Discussion

In a previous study, we made an analogy between OHSS and IAH syndrome documenting the importance of dynamic monitoring of IAP, AsI and OV. All these parameters were significantly associated with the OHSS severity [8]. In the present research, we studied the women’s anthropometric data and their relationship with OHSS severity.

All OHSS management guidelines emphasize the importance of daily monitoring of weight and WC in women and simply state the fact that the severity of OHSS increases with increasing these parameters [5, 10–12]. In our work, we did not observe significant differences in weight, BMI, HC or WC between moderate, severe and critical OHSS groups. Our data are consistent with those by Ma et al., who noted that increasing BMI is not a risk factor for OHSS severity [15]. Malbrain et al., when examining patients in intensive care, also stated that there was no significant correlation between abdominal circumference and IAP level [16].

It is a well-known that IAP is determined by two elements—the IAV and Cab [17]. The WC in women reflects approximate IAV, but not Cab and associated IAP. Women can have the same ascetic fluid amount, but different Cab, different possibilities for abdominal cavity accommodation and, as a result, different IAP. According to the World Society of Abdominal Compartment Syndrome (WSACS) experts, Cab plays a key role in understanding the negative effects of unadapted IAV on IAP and organ perfusion, although it is currently one of the most neglected parameters in critically ill patients [18]. Cab extension indicates a loss of abdominal wall elasticity, while a decrease in Cab means that the same change in IAV will result in a larger change in IAP.

Malbrain et al., in their fundamental work, studied the stages of changing in the abdominal shape in critically ill patients with IAH/ACS and revealed a change from an ellipse to a sphere with a maximum increase in IAP values. The authors described three phases of the ongoing processes: the reshaping, stretching, and pressurisation phases [19].

In the presented study, we obtained similar results. In the absence of significant intergroup differences in WC, the median APD in the moderate OHSS group was significantly lower than in the severe and critical OHSS group. Obviously, with the progression of ascites, APD increases most of all. The APD/TS ratio progressively increased and was highest at the most symptomatic stage of OHSS (Fig. 1). When the ratio APD/TS is approaching to 1, i.e. when the transverse and anteroposterior dimensions became equal, the abdomen took the sphere form with the transition from severe to critical OHSS. No significant difference in the APD/TS between mild and moderate OHSS can be explained by the fact that in moderate form there is a small amount of ascitic fluid with a sufficient elasticity reserve of the anterior abdominal wall and APD, as well as TS change insignificantly. Also, between severe and critical OHSS, there was no significant difference in the APD/TS. It can be due to the fact, that in severe form with exhaustion of abdominal stretching allowance, even a small addition of ascitic fluid slightly changes both, APD and TS, but causes an exponential increase in IAP with the transition to critical OHSS. Correlation analysis also confirmed a significant positive correlation between APD/TS and OHSS severity markers, where the strongest positive correlation was between APD/TS and IAP.

It can be assumed that women with severe OHSS had an initially lower Cab compared with mild OHSS, and an increase in additional IAV with limited Cab led to a progressive IAP increase. Unfortunately, Cab measurement and estimation are difficult at the patient’s bedside and can only be done in a case of change (removal or addition) in IAV [20]. This limitation also applies to IAV, which can be assessed by three-dimensional ultrasound, water-suppressed magnetic resonance imaging and computed tomography [19]. These are complex and expensive techniques which have not yet gained access to widespread clinical practice. Weak inverse correlation of APD/TS with WC seems illogical, although it can be explained by the fact that with increasing severity of OHSS, the median WC and BMI in the groups decreased, and the median Height increased (Additional file 2: Table S2). Thus, it can be stated that, asthenic type of constitution prevailed in the groups with severe and critical OHSS. The obtained results are consistent with the literature data, where asthenic habitus is indicated as one of the leading risk factors for the OHSS development [2, 10].

In a study assessing the IAV physiology during pregnancy, the authors confirm that the IAV capacity and the tensile properties of pregnant women's abdominal wall can be predicted by the dynamics of the anteroposterior and transverse abdominal diameters [21]. It should be pointed out that the current clinical guidelines represent pregnancy as a chronic compensated state of IAP, where the abdominal wall slowly stretches, its Cab gradually increases, and the pregnant woman has time to adapt to slowly increasing IAP levels [22]. Whereas OHSS is a dynamic condition, a rapid increase in volume and/or pressure exceeds Cab, because there is no time for tissue adaptation and moderate OHSS can progress to severe OHSS within a few hours [6]. Many authors confirm that in such cases, paracentesis is the single most important treatment modality for life-threatening OHSS which isn’t controlled by medical therapy [23–26]. Having the absence of the ability to measure IAP and Cab, the dynamics of the APD/TS ratio can be a surrogate indicator of the IAH degree, IAV increase, reserve capabilities of the abdominal wall’s extensibility and can help in establishing indications for timely performed paracentesis.

## Conclusions

The APD/TS ratio and its dynamics are important markers of OHSS severity. The APD/TS ratio increases progressively, reaching the highest values in the most symptomatic stage of OHSS.

IAP showed the strongest positive correlation with the APD/TS ratio; however, significant correlations were also found between APD/TS and AsI and OV.

When the ratio APD/TS is approaching to 1, and the anteroposterior and transverse abdominal dimensions become equal, the abdomen changes from an ellipse to a sphere, the reserve of abdominal wall stretching is depleted, and IAP exponential growth is observed with the transition from severe to critical OHSS. The APD/TS monitoring can be a method of indirectly controlling IAP, Cab and IAV reserve, without using complex and expensive techniques. The inclusion of APD/TS monitoring in the standard for the management of OHSS might be useful in specifying the severity and timely initiation of treatment, including methods to reduce IAP, prevent further organ dysfunction, and avoid the transition to a more severe stage of IAH and ACS. Finally, in the absence of IAP monitoring capabilities, the APD/TS ratio in conjunction with clinical and laboratory data might be an additional tool for indication for paracentesis.

## Supplementary Information

**Additional file 1: Table S1.** Baseline patients’ characteristics.

**Additional file 2: Table S2.** Clinical and laboratory data according to the severity of ovarian hyperstimulation syndrome.

**Additional file 3: Table S3.** Anthropometric markers according to the degree of severity of the ovarian hyperstimulation syndrome.

## Data Availability

The study was registered at the ISRCTN registry; identifier: ISRCTN66235250, http://www.isrctn.com/ISRCTN66235250 and https://doi.org/10.1186/ISRCTN66235250; Data are available at: Castelo-Branco, Camil (2021), “Severity Markers in Women with Ovarian Hyperstimulation Syndrome”, Mendeley Data, V1, https://doi.org/10.17632/ryhtps673s.1.

## Abbreviations

ACS: Abdominal compartment syndrome
APD: Anteroposterior diameter of the abdomen
AsI: Ascites index
Cab: Compliance of the abdominal wall
IAH: Intra-abdominal hypertension
IAP: Intra-abdominal pressure
IAV: Intra-abdominal volume
OHSS: Ovarian hyperstimulation syndrome
OV: Ovarian volume
TS: Transverse diameter of the abdomen
