# Supplementary material for: Are anthropometric data a tool for determining the severity of OHSS? Yes, it could be!
Source: BMC Womens Health. 2022 May 10;22:155. doi: 10.1186/s12905-022-01701-5 (PMC9092801; doi:10.1186/s12905-022-01701-5)
Supplement: Supplementary file 1 — Additional file 1: Table S1. Baseline patients’ characteristics. [file 12905_2022_1701_MOESM1_ESM.docx]

**Table S1.** **General patient characteristics.**

| Total number of patients | 76 |
| --- | --- |
| Age, years | 28 [IQR, 22–32] |
| Long protocol with GnRH-a, N (%) | 11 (14) |
| Short protocol with GnRH-a, N (%) | 6 (8) |
| Protocol with GnRH-ant, N (%) | 59 (78) |
| PCOS history, N (%) | 18 (24) |
| Oligomenorrhea, N (%) | 25 (31) |
| Pregnancy, N (%) | 73 (95) |
| Hospital stay, days | 8 [IQR, 4–13] |
| Primary/secondary infertility, N (%) | 49 (64)/27 (36) |
| Time between LMP and symptoms, days | 26 [IQR, 14–30] |
| Mild OHSS, N (%) | 25 (33) |
| Moderate OHSS, N (%) | 25 (33) |
| Severe OHSS, N (%) | 21 (28) |
| Critical OHSS, N (%) | 5 (6) |

Data are presented as median [interquartile range]. GnRH-a=gonadotropin-releasing hormone agonist; GnRH-ant=gonadotropin-releasing hormone antagonist; N=number of patients; PCOS=polycystic ovary syndrome; LMH=last menstrual period.
