# Supplementary material for: Are anthropometric data a tool for determining the severity of OHSS? Yes, it could be!
Source: BMC Womens Health. 2022 May 10;22:155. doi: 10.1186/s12905-022-01701-5 (PMC9092801; doi:10.1186/s12905-022-01701-5)
Supplement: Supplementary file 2 — Additional file 2: Table S2. Clinical and laboratory data according to the severity of ovarian hyperstimulation syndrome. [file 12905_2022_1701_MOESM2_ESM.docx]

**Table S2. Clinical and laboratory data according to the severity of ovarian hyperstimulation syndrome.**

| **Indicator** | **The severity of ovarian hyperstimulation syndrome** | | | | **P value** |
| --- | --- | --- | --- | --- | --- |
|  | **Mild**  (n = 25) | **Moderate**  (n = 25) | **Severe**  (n = 21) | **Critical**  (n = 5) |  |
| **Clinical parameters** |  | | | | |
| Intra-abdominal pressure (IAP), mmHg | 4.0  [3.0 – 6.5] | 12.0  [11.0 – 13.0] | 16.0  [14.5 – 18.0] | 25.0  [24.0 – 27.0] | *p*<0.001 |
| Ascites index (AsI), mm |  | 197  [140 – 235] | 285  [276 – 312] | 320  [310 – 346] | *p*<0.001 |
| Ovarian volumes (OV), ml | 307  [132 – 392] | 500  [441 – 561] | 578  [533 – 611] | 600  [487 – 704] | *p*<0.001 |
| **Blood cells & coagulation** |  | | | | |
| Hematocrit, % | 37.0  [34.2 – 39.0] | 42.0  [41.0 – 43.6] | 49.4  [46.4 – 53.2] | 56.1  [55.9 – 57.2] | *p*<0.001 |
| White blood cell /ml | 8 700  [6 700 –11 800] | 12 100  [10 900–  13 900] | 19 000  [15 200–23 900] | 26 000  [25 800 – 26 300] | *p*<0.001 |
| Platelets, ml/^3^ | 247  [181 – 294] | 192  [169 – 283] | 320  [262 – 369] | 396  [380 – 420] | *p*<0.001 |
| Fibrinogen, g/L | 3.4  [2.8 – 3.9] | 3.8  [3.2 – 4.2] | 5.7  [4.9 – 6.3] | 7.0  [6.7 – 7.1] | *p*<0.001 |
| **Basic metabolic panel** |  |  |  |  |  |
| Glucose, mmol/L | 4.9  [4.1 – 5.2] | 4.7  [4.1 – 5.2] | 4.6  [3.9 – 5.2] | 7.9  [7.2 – 8.1] | *p*<0.005 |
| Albumin, g/L | 41.3  [38.9 – 44.0] | 37.5  [35.0 – 40.0] | 30.6  [27.6 – 34.6] | 21.4  [20.3 – 23.6] | *p*<0.001 |
| Urea, mmol/L | 4.3  [3.2 – 5.1] | 5.2  [4.3 – 6.9] | 6.4  [5.7 – 7.1] | 7.8  [7.8 – 8.1] | *p*<0.001 |
| Creatinine, mmol/L | 78  [67 – 84] | 72  [69 – 81] | 84  [78 – 92] | 134  [127 – 137] | *p*<0.001 |
| Total bilirubin, mmol/L | 11.7  [9.3 – 16.3] | 12.7  [10.4 – 16.2) | 15.2  [12.7 – 19.0] | 19.4  [19.3 – 20.5] | *p*<0.001 |
| Aspartate aminotransferase, U/L | 21  [13 – 29] | 26  [17 – 32] | 44  [38 – 48] | 61  [59 – 68] | *p*<0.001 |
| Alanine aminotransferase, U/L | 19  [14 – 24] | 19  [15 – 25] | 29  [26 – 34] | 44  [43 – 49] | *p*<0.001 |
| Potassium, mmol/L | 4.1  [3.7 – 4.8] | 4.3  [3.8 – 4.8] | 5.0  [4.8 – 5.3] | 5.3  [4.9 – 5.5] | *p*<0.001 |
| Sodium, mmol/L | 140  [137 – 143] | 137  [136 – 140] | 135  [134 – 141] | 132  [131 – 133] | *p*<0.001 |

Data are presented as median [interquartile range]. Differences between groups were assessed with Kruskal-Wallis test.
