# Supplementary material for: Are anthropometric data a tool for determining the severity of OHSS? Yes, it could be!
Source: BMC Womens Health. 2022 May 10;22:155. doi: 10.1186/s12905-022-01701-5 (PMC9092801; doi:10.1186/s12905-022-01701-5)
Supplement: Supplementary file 3 — Additional file 3: Table S3. Anthropometric markers according to the degree of severity of the ovarian hyperstimulation syndrome. [file 12905_2022_1701_MOESM3_ESM.docx]

**Table S3. Anthropometric markers according to the degree of severity of the ovarian hyperstimulation syndrome**

| **Indicator** | **The severity of ovarian hyperstimulation syndrome** | | | | **P value** |
| --- | --- | --- | --- | --- | --- |
|  | **Mild**  (n = 25) | **Moderate**  (n = 25) | **Severe**  (n = 21) | **Critical**  (n = 5) |  |
| Height, cm | 167  [162 – 169] | 165  [162 – 173] | 165  [161 – 168] | 174  [169 – 176] | ns |
| Weight, kg | 57.0  [52.0 – 60.0] | 56.0  [53.0 – 63.5] | 54.0  [52.0 – 58.0] | 53.4  [53.0 – 58.6] | ns |
| Body mass index (BMI) | 20.7  [19.4 – 22.1] | 20.6  [20.2 – 21.2] | 20.3  [20.1 – 20.8] | 17.6  [17.2 – 20.6] | ns |
| Hip circumference (HC), cm | 95  [91 – 98] | 95  [92 – 97] | 94  [89 – 97] | 94  [89 – 96] | ns |
| Waist circumference (WC), cm | 89  [85 – 93] | 87  [83 – 92] | 86  [84 – 87] | 84  [83 – 86] | ns |
| Anteroposterior diameter of the abdomen (APD), cm | 16  [15 – 19] | 19  [16 – 24] | 24  [23 – 27] | 26  [24 – 28] | *p*<0.001 |
| Transverse diameter of the abdomen (TS), cm | 32  [26 – 36] | 28  [24 – 36] | 28  [25 – 30] | 23  [23 – 26] | *p*<0.01 |
| APD/TS | 0.55  [0.44 – 0.64] | 0.65  [0.61 – 0.70) | 0.87  [0.80 – 0.93] | 1.04  [1.04 – 1.13] | *p*<0.001 |

Data are presented as median [interquartile range]. Differences between groups were assessed with Kruskal-Wallis test. ns=no significant difference.
